# MicroRNA and mRNA Interaction Network Regulates the Malignant Transformation of Human Bronchial Epithelial Cells Induced by Cigarette Smoke

**DOI:** 10.3389/fonc.2019.01029

**Published:** 2019-10-09

**Authors:** Jin Wang, Xiao-fan Yu, Nan Ouyang, Shiyu Zhao, Haiping Yao, Xifei Guan, Jian Tong, Tao Chen, Jian-xiang Li

**Affiliations:** Department of Toxicology, School of Public Health, Medical College of Soochow University, Suzhou, China

**Keywords:** cigarette smoke, BEAS-2B, miRNA-mRNA network, lung cancer, The Cancer Genome Atlas

## Abstract

This study analyzes the correlation and interaction of miRNAs and mRNAs and their biological function in the malignant transformation of BEAS-2B cells induced by cigarette smoke (CS). Normal human bronchial epithelial cells (BEAS-2B) were continuously exposed to CS for 30 passages (S30) to establish an *in vitro* cell model of malignant transformation. The transformed cells were validated by scratch wound healing assay, transwell migration assay, colony formation and tumorigenicity assay. The miRNA and mRNA sequencing analysis were performed to identify differentially expressed miRNAs (DEMs) and differentially expressed genes (DEGs) between normal BEAS-2B and S30 cells. The miRNA-seq data of lung cancer with corresponding clinical data obtained from TCGA was used to further identify lung cancer-related DEMs and their correlations with smoking history. The target genes of these DEMs were predicted using the miRDB database, and their functions were analyzed using the online tool “Metascape.” It was found that the migration ability, colony formation rate and tumorigenicity of S30 cells enhanced. A total of 42 miRNAs and 753 mRNAs were dysregulated in S30 cells. The change of expression of top five DEGs and DEMs were consistent with our sequencing results. Among these DEMs, eight miRNAs were found dysregulated in lung cancer tissues based on TCGA data. In these eight miRNAs, six of them including miR-96-5p, miR-93-5p, miR-106-5p, miR-190a-5p, miR-195-5p, and miR-1-3p, were found to be associated with smoking history. Several DEGs, including *THBS1, FN1, PIK3R1, CSF1, CORO2B*, and *PREX1*, were involved in many biological processes by enrichment analysis of miRNA and mRNA interaction. We identified the negatively regulated miRNA-mRNA pairs in the CS-induced lung cancer, which were implicated in several cancer-related (especially EMT-related) biological process and KEGG pathways in the malignant transformation progress of lung cells induced by CS. Our result demonstrated the dysregulation of miRNA-mRNA profiles in cigarette smoke-induced malignant transformed cells, suggesting that these miRNAs might contribute to cigarette smoke-induced lung cancer. These genes may serve as biomarkers for predicting lung cancer pathogenesis and progression. They can also be targets of novel anticancer drug development.

## Introduction

Lung cancer is one of the most common carcinomas in men and women around the world. It is the first and second leading cause of cancer-related deaths in men and women, respectively (1, 2). There were 2.09 million new lung cancer cases and 1.76 million lung cancer deaths, which accounts for about 18.4% of all cancer deaths around the world in 2018 (3). The incidence of lung cancer is closely associated with cigarette smoking (2, 4). The risk of developing lung cancer in smokers is nearly ten times higher than that in non-smokers (5, 6). However, it is still not clear how normal lung epithelial cells become cancerous change in cigarette smokers.

It is well-known that the initiation and development of lung cancer are associated with abnormal expression of oncogenes and tumor suppressor genes. Numerous evidence suggested that the change in gene expression, which affects the occurrence and progression of tumors is closely related to epigenetic modification (7). Epigenetic modification could be DNA methylation, microRNAs (miRNAs), histone modifications, and nucleosome remodeling. These modifications are independent but could interact with each other to regulate gene expression (8). Epigenetic disruptions could promote the acquisition of a cancerous phenotype and aggressive behavior in lung cancer cells as well as primary or acquired resistance to treatment (9).

MiRNAs are highly conserved non-coding RNAs and consist of 18–24 nucleotides (nt) that are involved in the post-transcriptional regulation of gene (10). An individual miRNA is able to regulate many different transcripts. It is also believed that miRNAs can regulate more than one in three coding RNAs in the genome (11). MiRNAs participate in many vital biological processes through pairing with target mRNAs and regulating their expression (12, 13). The imbalance of miRNAs is usually associated with the progression and suppression of cancer, suggesting that miRNAs may play important roles as oncogenes or tumor suppressors (14).

The rapid development of high-throughput next-generation sequencing technology made it possible to identify changes in single bases in coding sequences of specific genes during lung tumorigenesis. A meticulous and thorough analysis of these data identified various vital genes and signaling pathways related to the tumor resulting in a better understanding of the mechanism of occurrence, development, and prognosis of lung cancer. Using novel technology and bioinformatics analysis, The Cancer Genome Atlas (TCGA, http://cancergenome.nih.gov/) project has previously identified panels of genetic mutations contributed to or were associated with the cause of a variety of cancers (15). Recently, the TCGA had shown studies on lung adenocarcinoma (LUAD) and squamous cell carcinomas (LUSC) at the molecular level (16, 17).

The aim of this study is to analyze the correlation and regulating mechanism of the regulatory network of miRNAs and mRNAs during carcinogenesis. An *in vitro* cell model of malignant transformation was established by exposing normal lung epithelial cells BEAS-2B to cigarette smoke (CS). Using high-throughput sequencing analysis, we analyzed the miRNA and mRNA expression profile in BEAS-2B cells with or without CS exposure. The differential expression miRNAs (DEMs) and differentially expressed genes (DEGs) were selected, and the integrative miRNA-mRNA network was analyzed. We identified some critical genes involved in carcinogenesis. This study will provide potential target candidates for novel drug development.

## Methods

### Preparation of Malignantly Transformed Cells

The CS-exposed malignant transformed cell model was established as described previously. Briefly, exponentially growing BEAS-2B cells were plated onto transwell membrane (Corning, USA) with 1 × 10^5^ in a single well (18). CS was produced using an automatic smoking machine, and the CS was pumped into an inhalation exposure chamber. Cells were directly exposed to CS for 10 min every day at a smoke concentration of 20%. This procedure was continued until the cells reached 30 passages and named S30 cells (18).

### Scratch Wound Healing Assay

The normal BEAS-2B cells and S30 cells (2 × 10^5^) were seeded into 6 well plates and cultured at 37 °C. Cells were allowed to grow up to 100% confluence and a scratch was made in the plate using a P10 pipette. The cells were cultured in fresh serum-free DMEM medium. Images were collected at 0 and 24 h under an inverted microscope (Olympus, Germany) and quantitatively analyzed with NIH ImageJ software.

### Transwell Migration Assay

The normal BEAS-2B cells and S30 cells (2 × 10^5^) were seeded in the upper chambers (pore size, 8 μm) of the 6 well plate (Corning, USA) in 1 ml serum-free medium. The lower chambers were filled with 2 ml complete medium with 10% FBS, and the plate was incubated under standard conditions for 24 h. At the end of incubation, after removing the cells in the upper surface of the membrane with a cotton swab, cells in the lower chamber were fixed with methanol and stained with 0.5% crystal violet solution. The images were taken with an inverted microscope (Olympus, Germany) and analyzed using NIH ImageJ software.

### Colony Formation Assay

1 × 10^3^ normal BEAS-2B cells and S30 cells were plated in 0.35% agarose on top of a 0.7% agarose base supplemented with complete medium. The medium was renewed every 2–3 days. The colonies were stained with 0.5% crystal violet (Sigma, USA) for 20 min at room temperature. The colony formation rate was calculated by the following equation: colony formation rate = the number of colonies/number of seeded cells × 100%.

### Tumorigenicity Assay

Five-week-old male BALB/c-nude mice of SPF grade were purchased from Beijing Vital River Laboratory Animal Technology Company Limited (Beijing, China). All nude mice were housed in the Laboratory Animal Center Soochow University. The animal experiment protocol was approved by the Laboratory Animal Ethics Committee of Experiment Animal Center of the Soochow University (Suzhou, China). Approximately 5 × 10^6^ normal BEAS-2B cells or S30 cells were injected subcutaneously into the right flank of male BALB/c-nude mice (5 mice were used for BEAS-2B cells injection and 10 mice for S30 cells injection). Animals were euthanized 45 days after injection, and tumors were collected and photographed.

### RNA Extraction and Sequencing

Total cellular RNA was extracted from S30 and normal BEAS-2B cells using TRIzol reagent (Invitrogen, USA) according to the manufacturer's protocol. Small RNA sequencing was performed on the Illumina Hiseq 2500 platform (Illumina, San Diego, CA). NEBNext® Multiplex Small RNA Library Prep Set for Illumina® (NEB, USA.) was used to prepare the small RNA sequencing library. To determine the known and novel miRNAs, unique clustered reads were aligned with the reference genome and database obtained from miRBase 20.0 (http://www.mirbase.org/). The miRDeep2 algorithm was used to predict novel miRNA precursors. The expression levels were estimated by transcript per million (TPM) and mRNA sequencing was performed on the Illumina HiSeq 4000 platform. The Illumina TruSeq RNA kit was used for preparing the mRNA sequencing library. The mRNAs with expression profiles that differed between the samples were normalized as fragments per kilobase of transcript per million mapped reads (FKPM). The DEGSeq package was used to analyze the differential expressed miRNAs (DEMs) or mRNAs (DEGs). *P*-value < 0.05 and |log2 (foldchange)| ≥ 1 were regarded as the threshold of significantly differential expression.

### Data Source and Processing

The NSCLC (LUAD and LUSC) miRNA-Seq datasets and related clinicopathology information were obtained from the Xena (https://xena.ucsc.edu). The LUAD miRNA expression data included a total of 493 samples consisting of 448 LUAD samples and 45 normal adjacent samples. The LUSC miRNA expression data included a total of 380 samples comprising 336 LUSC samples and 44 normal adjacent samples. The limma package was used to identify the differential expressed miRNAs in LUAD and LUSC when compared with corresponding normal adjacent samples. The differentially expressed miRNAs were defined by a threshold of *p*-value < 0.05 and |log2 (foldchange)| ≥ 1.

### Real-Time Quantitative PCR

A total of 1.5 μg RNA isolated from each sample was reversely transcribed into complementary DNA (cDNA) using Revert Aid First Strand Complementary DNA Synthesis Kit (for mRNA detecting, Thermo, USA) or Mir-X™ miRNA First-Strand Synthesis Kit (for miRNA detecting, Clontech Laboratories, USA) according to the manufacturer's instructions. Quantitative PCR (qPCR) was performed using NovoScript® SYBR Two-Step qRT-PCR Kit (novoprotein, China) with a QuantStudioTM 6 Flex qRT-PCR system (USA). The internal control for the quantitive analysis of miRNA and mRNA were U6 and GAPDH, respectively. The primer used for qPCR were listed in Table 1.

**Table 1:** Primers used in this study.

**Symbol**		**Sequence**
miR-106b-5p	Forward	5′-TAAAGTGCTGACAGTGCAGAT-′3
miR-589-5p	Forward	5′-TGAGAACCACGTCTGCTCTGAG-′3
miR-96-5p	Forward	5′-TTTGGCACTAGCACATTTTTGCT-′3
miR-181a-5p	Forward	5′-AACATTCAACGCTGTCGGTGAGT-′3
miR-361-3p	Forward	5′-TCCCCCAGGTGTGATTCTGATTT-′3
IGFBP3	Forward	5′-AGAGCACAGATACCCAGAACT-′3
	Reverse	5′-GGTGATTCAGTGTGTCTTCCATT-′3
KRT17	Forward	5′-GCCGCATCCTCAACGAGAT-′3
	Reverse	5′-CGCGGTTCAGTTCCTCTGTC-′3
FAM129A	Forward	5′-GCCTGGAAGGAACGATCCG-′3
	Reverse	5′-GGCCACCATCGCTTTGATCTT-′3
FLNC	Forward	5′-GCTCGTGTCCATAGACAGCAA-′3
	Reverse	5′-CTGGGGCACCTTGTTCTGG-′3
TIE1	Forward	5′-ACGACCATGACGGCGAATG-′3

*Reverse primers of miRNAs and U6 primers are provided by Mir-X™ miRNA First-Strand Synthesis Kit*.

### Analysis of Gene Expression and Smoking History

To validate the correlation between expression of miRNAs and patients' smoking history, all valid LUAD samples in the TCGA database were divided into four groups according to smoking history, including (1) lifelong non-smoker (*n* = 66); (2) current smokers (*n* = 104); (3) Current reformed smoker for >15 years (*n* = 116); (4) current reformed smoker for ≤15 years (*n* = 144). The expression of miRNAs in lung adenocarcinoma tissues of each group was compared.

### miRNA-mRNA Integrative Network

For identification of the candidate miRNA-mRNA network in smoking-induced malignant transformed cells, two separate steps were carried out. First, the target mRNAs of interest miRNAs were predicted through the miRDB database (http://mirdb.org/miRDB/). Second, the intersection of differently expressed genes and target genes was taken to screen the potential target genes of miRNAs in smoking-induced malignant transformed cells. These different expression target genes and miRNAs were used to construct the miRNA-mRNA regulation network through the Cytoscape software (V3.7.1, https://cytoscape.org).

### Enrichment Analyses

Metascape (http://metascape.org/gp/index.html) is an effective and efficient tool for experimental biologists to comprehensively analyze and interpret OMICs-based studies in the big data era (19). The database was used to perform the Gene Ontology (GO) and Kyoto Encyclopedia of Genes and Genomes (KEGG) pathway enrichment analysis, which is used to predict the potential biological functions of the overlapping genes of the DEGs and target genes.

### Statistical Analysis

SPSS 22.0 was used for statistical analysis. Values were presented as mean ± standard deviation (SD). Difference analysis between two groups was performed by using student *t*-test. A *p* < 0.05 was considered statistically significant. Correlation between differentially expressed miRNAs and predicted target mRNAs were calculated by Pearson's correlation. A *p* < 0.05 was regarded as statistically significant.

## Results

### CS-Induced Malignant Transformation in BEAS-2B Cells

**Figure 1:**
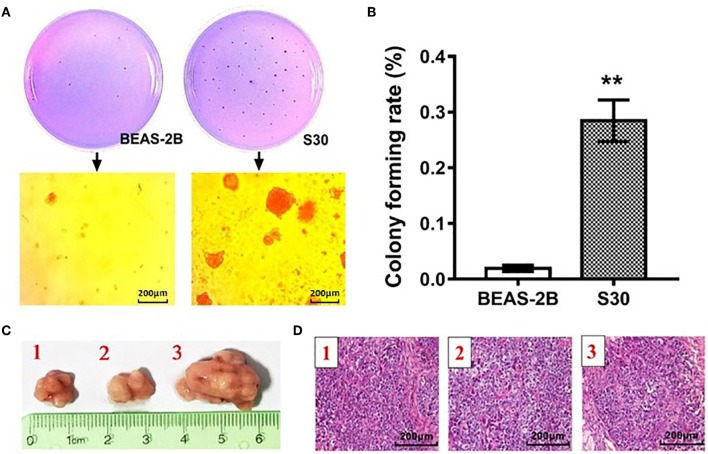
CS-induced malignant transformation in BEAS-2B cells *in vitro* and *in vivo*. **(A)** Representative photographs of the colony formation assay of the normal BEAS-2B cells and S30 cells. **(B)** Graph of soft agar colony forming rate of normal BEAS-2B cells and S30 cells. **p < 0.01 vs. BEAS-2B. **(C)** Photographs of tumors excised 45 days after injection of normal BEAS-2B cells and S30 cells into nude mice. **(D)** Representative HE staining histopathologic image of tumor tissues excised 45 days after injection of S30 cells into nude mice.

To validate the malignant change of S30 cells, the normal BEAS-2B cells and S30 cells were seeded in soft agar. As shown in Figure 1A, cells formed significantly more and bigger colonies in S30 cells compared to the normal BEAS-2B cells. Besides, colony formation rate in S30 cells was remarkably higher than in the normal BEAS-2B cells (Figure 1B). Moreover, the normal BEAS-2B cells and S30 cells were used to generate xenograft tumors in nude mice. Among the ten mice injected with S30 cells, 3 developed tumor tissue (Figures 1C,D). While no tumor was found in the five mice injected with normal BEAS-2B cells.

### CS Promoted the Migratory Ability of BEAS-2B Cells

**Figure 2:**
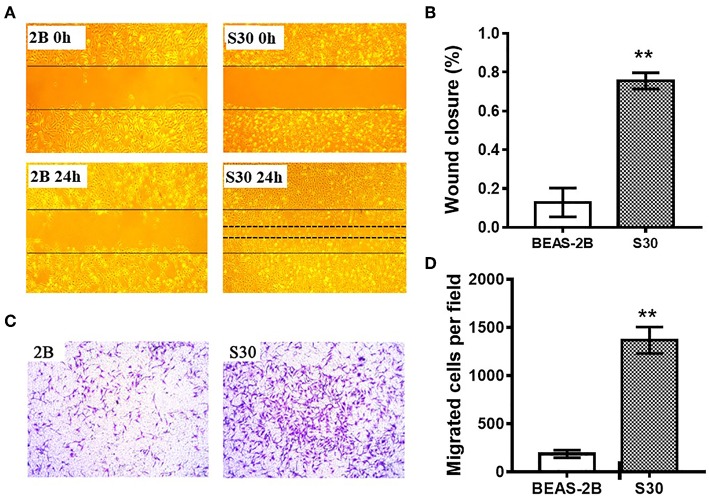
CS promoted the migratory ability of BEAS-2B cells. **(A)** Representative images of scratch wound healing assay of normal BEAS-2B cells and CS-induced malignant transformed cells (S30). **(B)** Graph of wound closure rate in scratch wound healing assay of normal BEAS-2B cells and S30 cells. **p < 0.01 vs. BEAS-2B. **(C)** Representative images of transwell assay of normal BEAS-2B cells and S30 cells. **(D)** Graph of migrated cells in transwell assay of normal BEAS-2B cells and S30 cells. **p < 0.01 vs. BEAS-2B.

To investigate the effect of CS in cell migration, we performed scratch wound healing and transwell cell migration assays. Scratch wound healing assay indicated that the migratory ability was significantly increased in S30 cells compared to the normal BEAS-2B cells (Figures 2A,B). As shown in Figures 2C,D, further transwell migratory assay demonstrated that the migrated cells were significantly increased in S30 cells compared to the normal BEAS-2B cells. These results suggested that long-term exposure to CS could promote the migratory ability of BEAS-2B cells.

### Differentially Expressed miRNAs Between S30 Cells and Normal BEAS-2B Cells

**Figure 3:**
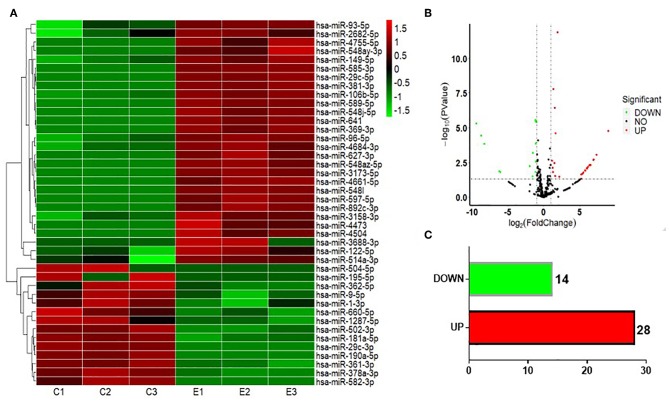
Graphical representation of the 42 miRNAs differentially expressed between S30 cells and normal BEAS-2B cells. **(A)** Heatmap of the 42 differentially expressed miRNAs (DEMs) between the CS-induced malignant transformed cells (S30) and normal BEAS-2B cells. The colors in the heatmap represent the normalized expression values, with low expression values being colored in shades of green and high expression values in shades of red. **(B)** Volcano plots were generated to visualize the distribution of DEMs between normal BEAS-2B and S30 cells. The top five most significantly dysregulated miRNAs are marked. **(C)** Counts of miRNAs upregulated or downregulated in S30 cells.

**Figure 4:**
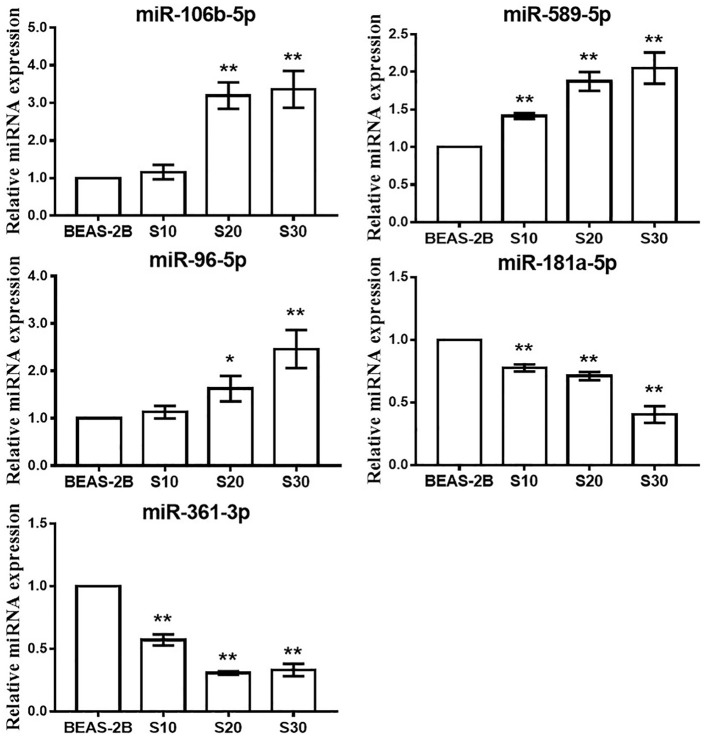
qPCR analysis of the top five most significantly dysregulated miRNAs in CS-exposed cells. Data are presented as relative fold induction compared with normal BEAS-2B cells. *p < 0.05, **p < 0.01 vs. BEAS-2B.

To test whether CS affects the miRNA-mRNA regulatory network in BEAS-2B cells, the miRNAs in normal BEAS-2B cells and S30 cells were quantitatively analyzed using small RNA sequencing. Compared with the normal BEAS-2B cells, the S30 cells showed dysregulation of 42 miRNAs that had significantly different expression levels with 2-fold change as a cut off (Figures 3A,B, Supplementary Table 1). Of these 42 miRNAs, 28 were upregulated (67%), and 14 were downregulated (33%) in the S30 cells (Figure 3C). The top five most significantly aberrant expression miRNAs are marked in the scatter plot; miR-106b-5p, miR-589-5p, and miR-96-5p were upregulated, and miR-181a-5p and miR-361-3p were downregulated (Figure 3B). The qPCR results of the top five miRNAs showed the increased miR-106b-5p, miR-589-5p, and miR-96-5p and decreased miR-181a-5p and miR-361-3p expression in S30 cells compared to normal BEAS-2B cells (Figure 4).

### Differentially Expressed mRNAs Between S30 Cells and Normal BEAS-2B Cells

**Figure 5:**
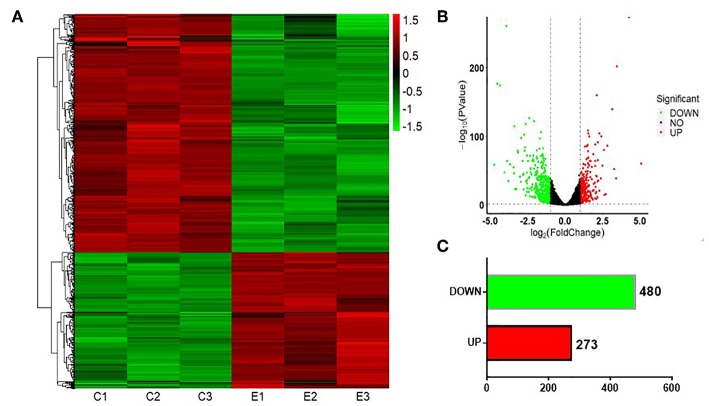
Graphical representation of the 753 mRNAs differentially expressed between S30 cells and normal BEAS-2B cells. **(A)** Heatmap of the 753 differentially expressed genes (DEGs) between the CS-induced malignant transformed cells (S30) and normal BEAS-2B cells. The colors in the heatmap represent the normalized expression values, with low expression values being colored in green and high expression values in red. **(B)** Volcano plots were generated to visualize the distribution of DEGs between normal BEAS-2B and S30 cells. The top five most significantly dysregulated genes are marked. **(C)** Counts of genes upregulated or downregulated in S30 cells.

**Figure 6:**
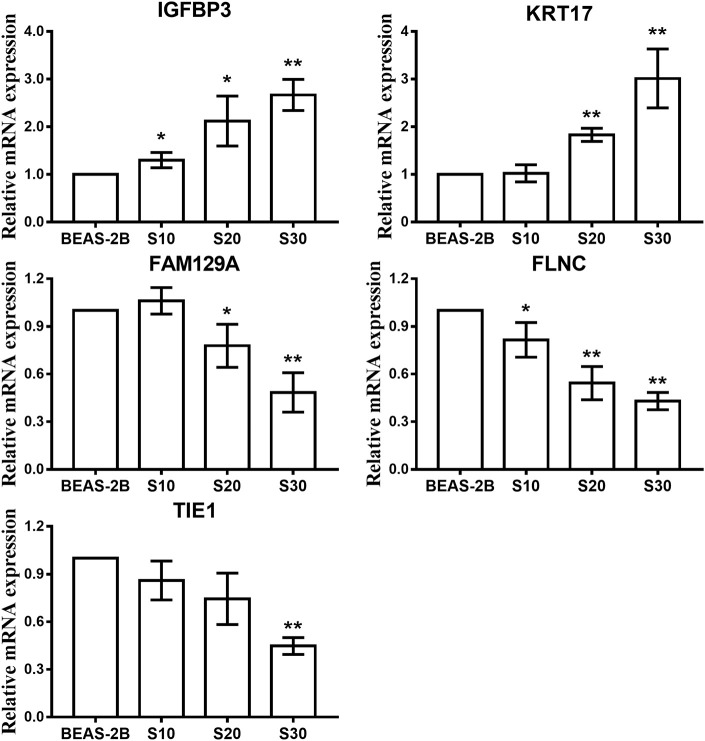
qPCR analysis of the top five most significantly dysregulated genes in CS-exposed cells. Data are presented as relative fold induction compared with normal BEAS-2B cells. *p < 0.05, **p < 0.01 vs. BEAS-2B.

Next, we investigated the expression patterns of mRNAs using transcriptome resequencing. Compared with the normal BEAS-2B cells, the S30 cells showed dysregulation of 753 mRNAs that had significantly different expression levels with 2-fold change as a cut off (Figures 5A,B). Of these 753 mRNAs, 273 were upregulated (36%), and 480 were downregulated (64%) in the S30 cells (Figure 5C). The top five most significantly dysregulated mRNAs are marked in the scatter plot; *IGFBP3* and *KRT17* were upregulated, and *FAM129A, FLNC*, and *TIE1* were downregulated (Figure 5B). The qPCR results of the top five mRNAs validated the increased *IGFBP3* and *KRT17* and decreased *FAM129A, FLNC*, and *TIE1* expression in S30 cells compared to normal BEAS-2B cells (Figure 6).

### Integrated Analysis of the DEMs in S30 Cells and Lung Cancer Samples

**Figure 7:**
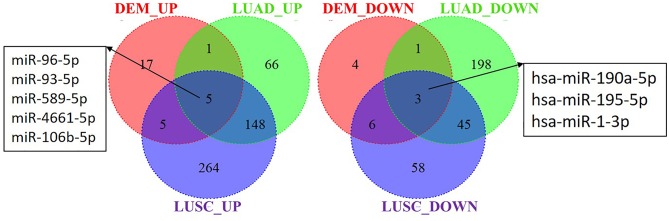
Identification of differential expressed miRNAs in S30 cells and lung cancer samples. DEM_UP/DEM_DOWN: up-regulated/ down-regulated miRNAs in S30 cells; LUAD_UP/LUAD_DOWN: up-regulated/ down-regulated miRNAs in LUAD samples; LUSC_UP/LUSC_DOWN: up-regulated/ down-regulated miRNAs in LUSC samples.

To explore whether the DEMs' expression is altered in lung cancer tissues, we analyzed the miRNAs sequencing data of lung cancer, including lung adenocarcinomas (LUAD) and squamous cell lung carcinomas (LUSC), in the TCGA database. A total of 8 miRNAs were found dysregulation in S30 cells, LUAD and LUSC samples with a similar tendency of change. Among these 8 miRNAs, 5 were upregulated (miR-96-5p, miR-93-5p, miR-589-5p, miR-4661-5p, and miR-106b-5p) and 3 were downregulated (miR-190a-5p, miR-195-5p, and miR-1-3p) in the three datasets (Figure 7).

### Association of miRNA Expression With Smoking History

Among the five up-regulated miRNAs, three miRNAs, including miR-96-5p, miR-93-5p, and miR-106-5p, showed a higher expression in current smoking LUAD patients when compared with the lifelong non-smokers (Table 2). Three of the screened down-regulated miRNAs, including miR-190a-5p, miR-195-5p, and miR-1-3p, showed lower expression in current smoking LUAD patients when compared with the lifelong non-smokers (Table 2). Moreover, miR-96-5p and miR-106b-5p are overexpressed in the current reformed smoker for >15 years, while miR-190a-5p has lower expression in the current reformed smoker for >15 years when compared with the lifelong non-smoker (Table 2).

**Table 2:** The expression of miRNAs in the LUAD patients with different smoking history.

**miRNAs**	**Significant**	**1 (*n* = 66)**	**2 (*n* = 104)**	**3 (*n* = 116)**	**4 (*n* = 144)**
miR-96-5p	UP	4.25 ± 0.74	4.58 ± 1.07*	4.39 ± 1.12	4.55 ± 1.04*
miR-93-5p	UP	11.70 ± 0.87	12.09 ± 0.87**	11.65 ± 0.86	11.79 ± 0.97
miR-589-5p	UP	6.66 ± 0.65	6.72 ± 0.79	6.26 ± 0.71**	6.49 ± 0.77
miR-4661-5p	UP	2.03 ± 0.71	2.14 ± 1.05	1.94 ± 0.84	2.10 ± 1.02
miR-106b-5p	UP	7.72 ± 0.65	8.21 ± 0.72**	7.75 ± 0.68	8.03 ± 0.72**
miR-190a-5p	DOWN	1.87 ± 0.77	1.52 ± 0.74**	1.74 ± 0.65	1.57 ± 0.56**
miR-195-5p	DOWN	5.15 ± 0.98	4.73 ± 0.84**	5.11 ± 0.82	5.02 ± 0.87
miR-1-3p	DOWN	3.37 ± 1.41	2.44 ± 1.25**	3.41 ± 1.34	3.04 ± 1.28

Lifelong non-smoker (<100 cigarettes smoked in Lifetime) = 1; Current smoker (includes daily smokers and non-daily smokers or occasional smokers) = 2; Current reformed smoker for > 15 years (> 15 years) = 3; Current reformed smoker for ≤ 15 years (≤15 years) = 4 (*p < 0.05 vs. non-smoker; **p < 0.01 vs. non-smoker).

### Integrative Analysis of Correlation of miRNA and mRNA in S30 Cells

**Figure 8:**
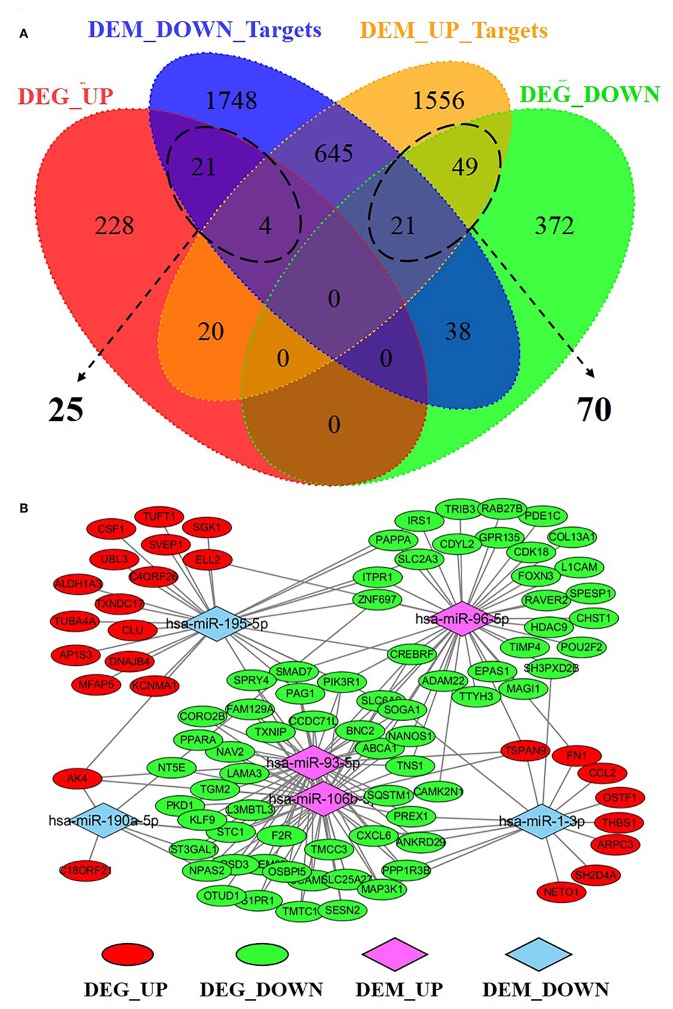
Integrative analysis of miRNA-mRNA regulatory network in S30 cells. **(A)** Venn Diagram depicting the distribution of negatively correlated miRNA-mRNA pairs within the four datasets. **(B)** Regulatory network for 95 negatively correlated miRNA-mRNA, including 25 DEG_UP/DEM_DOWN_Targets pairs and 70 DEG_DOWN/DEM_UP_Targets pairs. DEG_UP/DEG_DOWN: up-regulated/down-regulated genes in S30 cells; DEM_UP/DEM_DOWN: up-regulated/ down-regulated miRNAs in S30 cells.

To understand the potential functions of the smoking-related differentially expressed miRNAs, and to explore miRNA-mRNA interaction in S30 cells, we predicted the target genes of miRNAs and performed an intersection analysis with the gene expression data to identify genes that were inversely co-expressed with miRNAs. A total of 2,477 target genes of low-expressed miRNAs and 2,295 target genes of high-expressed miRNAs were screened by searching miRDB database. Consequently, 25 up-regulated genes and 70 down-regulated genes were found to have at least one negatively regulated miRNA-mRNA pair for smoking-related differentially expressed miRNAs (Figure 8A, Supplementary Table 2). The miRNAs-DEGs network was generated by Cytoscape software, as showed in Figure 8B.

### Enrichment Analysis of Correlation of miRNA and mRNA in S30 Cells

**Figure 9:**
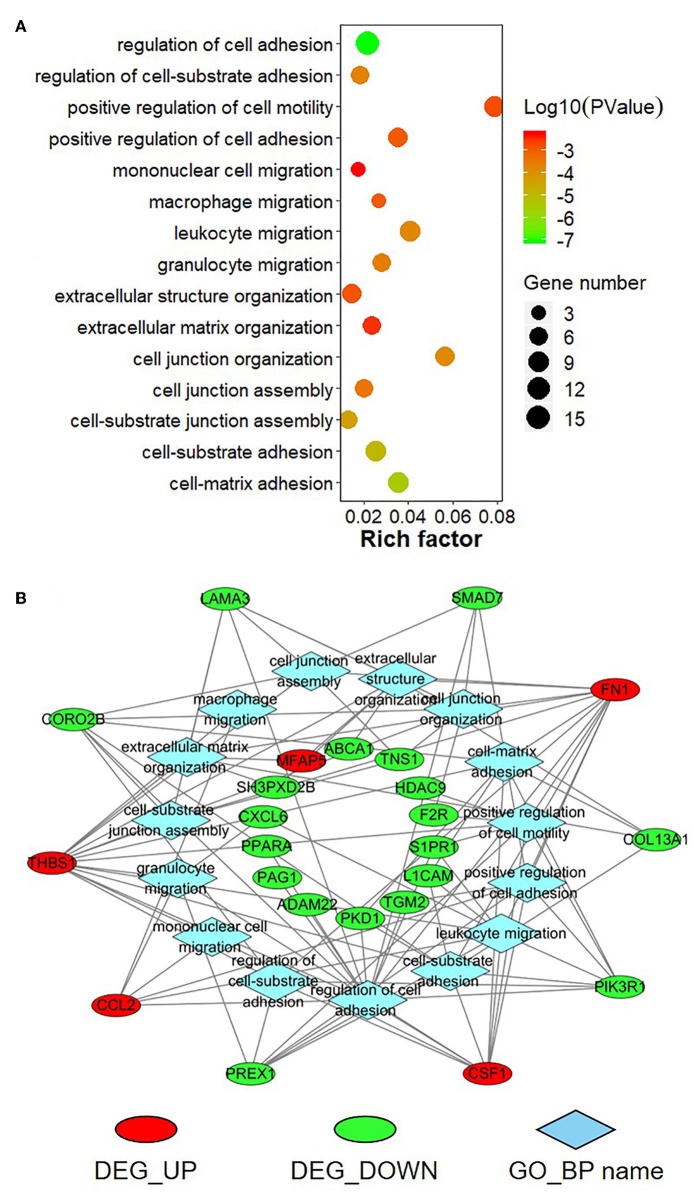
Gene Ontology (GO) enrichment analysis for negatively correlated miRNA-mRNA. **(A)** The top enriched GO terms are shown in the bubble chart. The Y-axis represents the biologic process GO terms, and the X-axis represents the rich factor (rich factor = amount of differentially expressed genes enriched in the GO term/amount of all genes in background gene set). The color and size of the bubble represent enrichment significance and the number of genes enriched in the GO term, respectively. **(B)** Network diagram of top enriched GO terms for the negatively correlated miRNA-mRNA. DEG_UP: up-regulated differentially expressed genes; DEG_Down: down-regulated differential expression genes; GO_BP: Gene Ontology of biological process.

**Figure 10:**
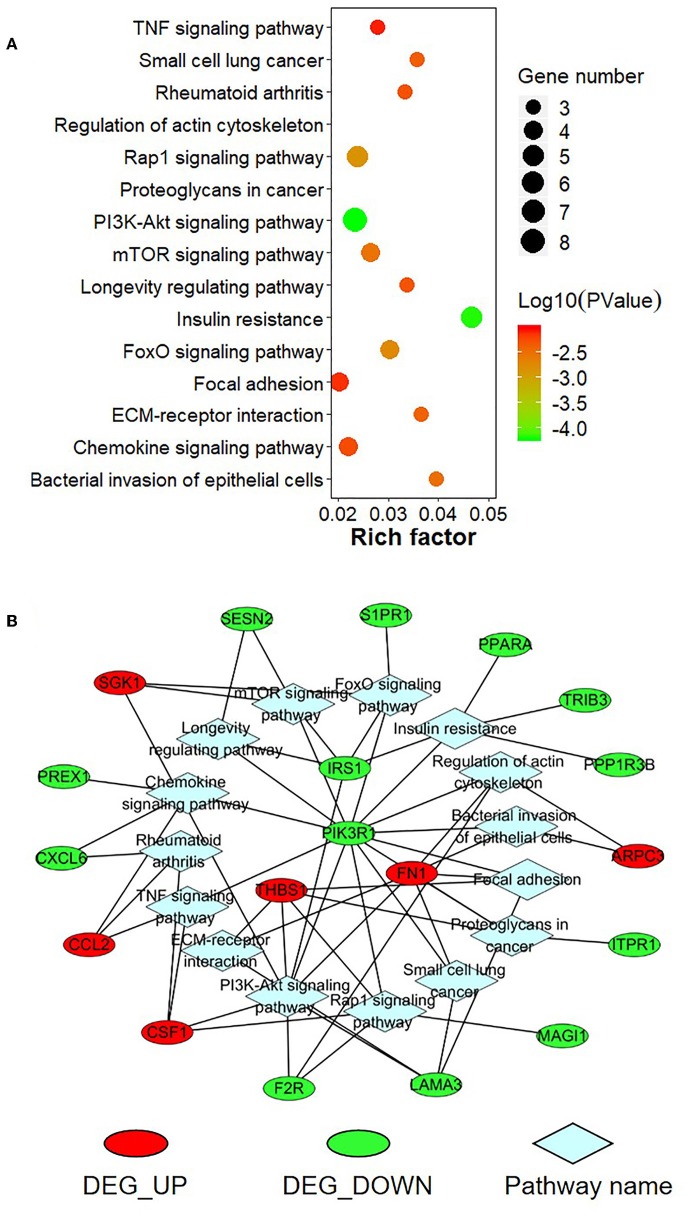
KEGG pathway enrichment analysis for negatively correlated miRNA-mRNA. **(A)** The top enriched KEGG pathways are shown in the bubble chart. The Y-axis represents the KEGG pathways, and the X-axis represents the rich factor (rich factor = amount of differential expressed genes enriched in the pathway/amount of all genes in the background gene set). The color and size of the bubble represent enrichment significance and the number of genes enriched in the pathway, respectively. **(B)** Network diagram of top enriched KEGG pathways for the negatively correlated miRNA-mRNA. DEG_UP: up-regulated differential expressed genes; DEG_Down: down-regulated differential expression genes.

To further explore the function of the negatively correlated miRNA-mRNA pairs, 95 up-regulated or down-regulated target genes in S30 cells were selected for mapping into the Metascape database and subjected to functional enrichment analysis. As shown in Figure 9A, GO analysis demonstrated that these target genes are associated with several cancer-related, especially tumor migration related GO terms, including “positive regulation of cell motility,” “regulation of cell adhesion,” “mononuclear cell migration,” “cell junction organization,” “extracellular structure organization” and so on. Among these enriched DEGs, several DEGs, including *THBS1, FN1, PIK3R1, CSF1, CORO2B*, and *PREX1*, were involved in many biologic processes which derived from enrichment analysis of negative miRNA-mRNA correlations (Figure 9B). Moreover, the KEGG pathway enrichment analysis suggested that these target genes were significantly correlated with “TNF signaling pathway,” “Small cell lung cancer,” “Rap1 signaling pathway,” “PI3K-Akt signaling pathway,” “mTOR signaling pathway,” “FoxO signaling pathway,” “Focal adhesion,” “ECM-receptor interaction,” and some other cancer-related pathways (Figure 10A). In particular, “Focal adhesion” and “ECM-receptor interaction” are closely related to cell migration. In addition, *THBS1, FN1, PIK3R1*, and *IRS1*, were involved in many KEGG pathways which derived from enrichment analysis of negative miRNA-mRNA correlations (Figure 10B).

## Discussion

There are nearly 1.3 billion cigarette smokers in the world, which leads to 5 million cancer related deaths every year (20). Cigarette-smoking is a notable risk factor for multiple pathologies (21–23), among them lung cancer takes the lead with smokers having a much higher risk than non-smokers. Our previous studies have suggested that chronic exposure to CS could induce malignant transformation of the human bronchial epithelial cell line (BEAS-2B) and tumorigenesis (18, 24). In recent years, studies have indicated that miRNAs play an essential role in tumor initiation, development, and metastasis as well as the cellular response to stress by modulating the expression of their target genes (25–27). In this study, we investigate the effect of chronic exposure to CS on the expression of miRNA and mRNA in BEAS-2B cells and further examined the interaction of miRNA and mRNAs.

Based on our high throughput sequencing data and the TCGA database analysis, we found significant dysregulation of 6 smoking-related miRNAs in S30 cells compared with normal BEAS-2B cells. Among these miRNAs, miR-190a is found downregulated in aggressive neuroblastoma (NBL). Overexpression of miR-190a contributed to the inhibition of tumor growth and prolonged the dormancy period of fast-growing tumors (28). A recent study showed that miR-190a could inhibit the metastasis of breast tumor by involving in estrogen receptor (ERα) signaling (29). miR-195 usually serves as a tumor suppressor in several cancer types, such as gastric cancer (30), renal cancer (31), cervical cancer (32), liver cancer (33), and osteosarcoma (34), and its downregulation was related to lymph node metastasis and advanced clinical stage (32). Similarly, miR-1 can regulate multiple behavior of the tumor cells, such as proliferation (35, 36), migration (37), apoptosis (38, 39), and metabolism (40). In addition, miR-106b and miR-93 are both the members of miR-106b~25 cluster, which have regarded as significant oncogenic drivers as well as potential biomarkers and therapeutic targets in various tumors (41–44). Moreover, Several studies have demonstrated that miR-96 could act as an oncogene (45–47) or tumor suppressor (48, 49) depending on the different types of cancer. Although these miRNAs have extensively been reported to be associated with many other kinds of cancer, their roles in lung cancer has yet been demonstrated.

Numerous studies have established the regulatory relationships between miRNA and mRNA expression (50, 51). CS-induced DEMs can bind to 3′UTR regions of several genes and down-regulate their expression, indicating that these miRNAs may contribute to the pathogenesis of smoking-related diseases. It has been reported that negatively regulated miRNA-mRNA pairs are significantly contributed to the initialization and development of different kinds of cancer (52–54). In order to further comprehend the roles of the miRNA-mRNA pairs in CS-induced lung cancer, we selected 95 dysregulated target mRNAs of the 6 CS-related miRNAs and found that they are involved in several cancer-related signaling pathways including TNF signaling pathway, Rap1 signaling pathway, PI3K-Akt signaling pathway, mTOR signaling pathway, FoxO signaling pathway, ECM-receptor interaction, and so on. Meanwhile, the GO enrichment analysis results indicated that these target genes were participated in a series of cell adhesion and migration biological processes, suggesting these miRNA-mRNA pairs related to the process of epithelial-mesenchymal transformation (EMT). EMT is considered to be an important regulator of metastasis by promoting the invasion and spread of tumor cells to distant organs (55). Among these enriched DEGs, *IRS1, PIK3R1, THBS1*, and *FN1* are related to more than 4 KEGG pathways. As an adaptor of the insulin growth factor-1 receptor, *IRS1* plays an essential role in cell growth and proliferation, primarily via the Akt pathway, and it was reported to be regulated by several miRNAs through direct or indirect action (56–59). Moreover, studies have demonstrated that *PIK3R1* was directly targeted by miR-127 (60), miR-21 (61), miR-155 (62), and some other miRNAs in different kinds of cancers. It's reported that the activity of phosphoinositide 3- kinase (PI3K) is activated by many oncogenes and the PI3K family members are involved in a serious of biological processes and the genesis and progression of various tumors (63). Thrombospondin 1 (*THBS1*) is a secreted protein with multiple biological functions (64), including a potent anti-angiogenic activity and activation of latent transforming growth factor beta (TGF-β) (65, 66). A recent study suggested that the expression of *THBS1* was modulated by multiple miRNAs (67). Moreover, it's reported that fibronectin 1 (*FN1*) is crucial to many cellular processes, including cell proliferation, adhesion, migration and differentiation (68, 69), and the expression of *FN1* was regulated by miR-1271 (70), miR-9 (71), and miR-206 (72). Similar to previous studies, we identified the negatively regulated miRNA-mRNA pairs in the CS-induced lung cancer, which were implicated in several cancer-related (especially EMT-related) biological process and KEGG pathways in the malignant transformation progress of lung cells induced by CS. Further study will be needed to explore the targeting relationships of these miRNAs and their target mRNAs and their possible roles on cancer-related molecular mechanisms for the development of novel targeted therapy for CS-induced lung cancer.

In conclusion, our study demonstrated that the expression profiles of miRNA and mRNA were significantly dysregulated in BEAS-2B cells with long-term exposure to CS. Smoking induced miRNAs are associated with EMT and carcinogenesis.

## Data Availability Statement

The datasets generated for this study can be found in the Sequence Read Archive (SRA) database (https://trace.ncbi.nlm.nih.gov/Traces/sra/) with identifier SRP182926 and SRP181756. The LUAD and LUSC datasets analyzed for this study can be obtained from UCSC Xena (https://xenabrowser.net/datapages/).

## Ethics Statement

The animal study was reviewed and approved by The Laboratory Animal Ethics Committee of Experiment Animal Center of the Soochow University. Written informed consent was obtained from the owners for the participation of their animals in this study.

## Author Contributions

JL conceived and designed the study. JW performed and analyzed the experiments. XY, NO, SZ, HY, and XG assisted to collect and analyze the data. JW wrote the manuscript. JT and TC were of immense help in the modification of the manuscript. All authors read and approved the final manuscript.

### Conflict of Interest

The authors declare that the research was conducted in the absence of any commercial or financial relationships that could be construed as a potential conflict of interest.

## References

[1] Ferlay J Soerjomataram I Dikshit R Eser S Mathers C Rebelo M. Cancer incidence and mortality worldwide: sources, methods and major patterns in GLOBOCAN 2012. Int J Cancer. (2015) 136:E359–386. 10.1002/ijc.29210 25220842

[2] Torre LA Bray F Siegel RL Ferlay J Lortet-Tieulent J Jemal A. Global cancer statistics, 2012. CA Cancer J Clin. (2015) 65:87–108. 10.3322/caac.21262 25651787

[3] Bray F Ferlay J Soerjomataram I Siegel RL Torre LA Jemal A. Global cancer statistics 2018: GLOBOCAN estimates of incidence and mortality worldwide for 36 cancers in 185 countries. CA Cancer J Clin. (2018) 68:394–424. 10.3322/caac.21492 30207593

[4] Thun M Peto R Boreham J Lopez AD. Stages of the cigarette epidemic on entering its second century. Tob Control. (2012) 21:96–101. 10.1136/tobaccocontrol-2011-050294 22345230

[5] Hecht SS. Tobacco smoke carcinogens and lung cancer. J Natl Cancer Inst. (1999) 91:1194–210. 10.1093/jnci/91.14.1194 10413421

[6] Shields PG. Molecular epidemiology of smoking and lung cancer. Oncogene. (2002) 21:6870–6. 10.1038/sj.onc.1205832 12362269

[7] Nebbioso A Tambaro FP Dell'Aversana C Altucci L. Cancer epigenetics: moving forward. PLoS Genet. (2018) 14:e1007362. 10.1371/journal.pgen.1007362 29879107 PMC5991666

[8] Portela A Esteller M. Epigenetic modifications and human disease. Nat Biotechnol. (2010) 28:1057–68. 10.1038/nbt.1685 20944598

[9] Duruisseaux M Esteller M. Lung cancer epigenetics: from knowledge to applications. Semin Cancer Biol. (2018) 51:116–28. 10.1016/j.semcancer.2017.09.005 28919484

[10] Kang SM Lee HJ. MicroRNAs in human lung cancer. Exp Biol Med. (2014) 239:1505–13. 10.1177/1535370214533887 24872439

[11] Bartel DP. MicroRNAs: target recognition and regulatory functions. Cell. (2009) 136:215–33. 10.1016/j.cell.2009.01.002 19167326 PMC3794896

[12] Lim LP Lau NC Garrett-Engele P Grimson A Schelter JM Castle J. Microarray analysis shows that some microRNAs downregulate large numbers of target mRNAs. Nature. (2005) 433:769–73. 10.1038/nature03315 15685193

[13] Valencia-Sanchez MA Liu J Hannon GJ Parker R. Control of translation and mRNA degradation by miRNAs and siRNAs. Genes Dev. (2006) 20:515–24. 10.1101/gad.1399806 16510870

[14] Esquela-Kerscher A Slack FJ. Oncomirs—microRNAs with a role in cancer. Nat Rev Cancer. (2006) 6:259–69. 10.1038/nrc1840 16557279

[15] Kandoth C McLellan MD Vandin F Ye K Niu B Lu C. Mutational landscape and significance across 12 major cancer types. Nature. (2013) 502:333–9. 10.1038/nature12634 24132290 PMC3927368

[16] Cancer Genome Atlas Research Network Comprehensive genomic characterization of squamous cell lung cancers. Nature. (2012) 489:519–25. 10.1038/nature11404 22960745 PMC3466113

[17] Cancer Genome Atlas Research Network Comprehensive molecular profiling of lung adenocarcinoma. Nature. (2014) 511:543–50. 10.1038/nature13385 25079552 PMC4231481

[18] Du H Sun J Chen Z Nie J Tong J Li J. Cigarette smoke-induced failure of apoptosis resulting in enhanced neoplastic transformation in human bronchial epithelial cells. J Toxicol Environ Health A. (2012) 75:707–20. 10.1080/15287394.2012.690088 22757675

[19] Zhou Y Zhou B Pache L Chang M Khodabakhshi AH Tanaseichuk O. Metascape provides a biologist-oriented resource for the analysis of systems-level datasets. Nat Commun. (2019) 10:1523. 10.1038/s41467-019-09234-6 30944313 PMC6447622

[20] Schembri F Sridhar S Perdomo C Gustafson AM Zhang XL Ergun A. MicroRNAs as modulators of smoking-induced gene expression changes in human airway epithelium. Proc Natl Acad Sci USA. (2009) 106:2319–24. 10.1073/pnas.0806383106 19168627 PMC2650144

[21] Blakely T Barendregt JJ Foster RH Hill S Atkinson J Sarfati D. The association of active smoking with multiple cancers: national census-cancer registry cohorts with quantitative bias analysis. Cancer Causes Control. (2013) 24:1243–55. 10.1007/s10552-013-0204-2 23580085

[22] Momi N Kaur S Rachagani S Ganti AK Batra SK. Smoking and microRNA dysregulation: a cancerous combination. Trends Mol Med. (2014) 20:36–47. 10.1016/j.molmed.2013.10.005 24238736 PMC3880642

[23] Krishnan AR Zheng H Kwok JG Qu Y Zou AE Korrapati A. A comprehensive study of smoking-specific microRNA alterations in head and neck squamous cell carcinoma. Oral Oncol. (2017) 72:56–64. 10.1016/j.oraloncology.2017.07.009 28797462 PMC5555648

[24] Huang H Ji Y Zhang J Su Z Liu M Tong J. Aberrant DNA methylation in radon and/or cigarette smoke-induced malignant transformation in BEAS-2B human lung cell line. J Toxicol Environ Health A. (2017) 80:1321–30. 10.1080/15287394.2017.1384156 29048996

[25] Cheng AM Byrom MW Shelton J Ford LP. Antisense inhibition of human miRNAs and indications for an involvement of miRNA in cell growth and apoptosis. Nucleic Acids Res. (2005) 33:1290–7. 10.1093/nar/gki200 15741182 PMC552951

[26] Chen B Li H Zeng X Yang P Liu X Zhao X. Roles of microRNA on cancer cell metabolism. J Transl Med. (2012) 10:228. 10.1186/1479-5876-10-228 23164426 PMC3563491

[27] Plaisier CL Pan M Baliga NS. A miRNA-regulatory network explains how dysregulated miRNAs perturb oncogenic processes across diverse cancers. Genome Res. (2012) 22:2302–14. 10.1101/gr.133991.111 22745231 PMC3483559

[28] Almog N Briggs C Beheshti A Ma L Wilkie KP Rietman E. *Transcription*al changes induced by the tumor dormancy-associated microRNA-190. Transcription. (2013) 4:177–91. 10.4161/trns.25558 23863200 PMC3977918

[29] Chu HW Cheng CW Chou WC Hu LY Wang HW Hsiung CN. A novel estrogen receptor-microRNA 190a-PAR-1-pathway regulates breast cancer progression, a finding initially suggested by genome-wide analysis of loci associated with lymph-node metastasis. Hum Mol Genet. (2014) 23:355–67. 10.1093/hmg/ddt426 24009311

[30] Wang J Li L Jiang M Li Y. MicroRNA-195 inhibits human gastric cancer by directly targeting basic fibroblast growth factor. Clin Transl Oncol. (2017) 19:1320–8. 10.1007/s12094-017-1668-4 28500362

[31] Wang K Sun Y Tao W Fei X Chang C. Androgen receptor (AR) promotes clear cell renal cell carcinoma (ccRCC) migration and invasion via altering the circHIAT1/miR-195-5p/29a-3p/29c-3p/CDC42 signals. Cancer Lett. (2017) 394:1–12. 10.1016/j.canlet.2016.12.036 28089832

[32] Zhong JY Yuan H Xu XQ Kong SF MicroRNA-195 inhibits cell proliferation, migration and invasion by targeting defective in cullin neddylation 1 domain containing 1 in cervical cancer. Int J Mol Med. (2018) 42:779–88. 10.3892/ijmm.2018.3660 29750306 PMC6034917

[33] Zhang H Zhou D Ying M Chen M Chen P Chen Z. Expression of long non-coding RNA (lncRNA) small nucleolar RNA host gene 1 (SNHG1) exacerbates hepatocellular carcinoma through suppressing miR-195. Med Sci Monit. (2016) 22:4820–9. 10.12659/msm.898574 27932778 PMC5167104

[34] Qu Q Chu X Wang P. MicroRNA-195-5p suppresses osteosarcoma cell proliferation and invasion by suppressing naked cuticle homolog 1. Cell Biol Int. (2017) 41:287–95. 10.1002/cbin.10723 28032380

[35] Reid JF Sokolova V Zoni E Lampis A Pizzamiglio S Bertan C. miRNA profiling in colorectal cancer highlights miR-1 involvement in MET-dependent proliferation. Mol Cancer Res. (2012) 10:504–15. 10.1158/1541-7786.MCR-11-0342 22343615

[36] Stope MB Stender C Schubert T Peters S Weiss M Ziegler P. Heat-shock protein HSPB1 attenuates microRNA miR-1 expression thereby restoring oncogenic pathways in prostate cancer cells. Anticancer Res. (2014) 34:3475–80. 24982356

[37] Xu L Zhang Y Wang H Zhang G Ding Y Zhao L. Tumor suppressor miR-1 restrains epithelial-mesenchymal transition and metastasis of colorectal carcinoma via the MAPK and PI3K/AKT pathway. J Transl Med. (2014) 12:244. 10.1186/s12967-014-0244-8 25196260 PMC4172896

[38] Nohata N Hanazawa T Kikkawa N Sakurai D Sasaki K Chiyomaru T. Identification of novel molecular targets regulated by tumor suppressive miR-1/miR-133a in maxillary sinus squamous cell carcinoma. Int J Oncol. (2011) 39:1099–107. 10.3892/ijo.2011.1096 21701775

[39] Yamasaki T Yoshino H Enokida H Hidaka H Chiyomaru T Nohata N. Novel molecular targets regulated by tumor suppressors microRNA-1 and microRNA-133a in bladder cancer. Int J Oncol. (2012) 40:1821–30. 10.3892/ijo.2012.1391 22378464

[40] Singh A Happel C Manna SK Acquaah-Mensah G Carrerero J Kumar S. Transcription factor NRF2 regulates miR-1 and miR-206 to drive tumorigenesis. J Clin Invest. (2013) 123:2921–34. 10.1172/JCI66353 23921124 PMC3696551

[41] Fang L Deng Z Shatseva T Yang J Peng C Du WW. MicroRNA miR-93 promotes tumor growth and angiogenesis by targeting integrin-beta8. Oncogene. (2011) 30:806–21. 10.1038/onc.2010.465 20956944

[42] Li F Liu J Li S. MicorRNA 106b approximately 25 cluster and gastric cancer. Surg Oncol. (2013) 22:e7–10. 10.1016/j.suronc.2013.01.003 23510949

[43] Li N Miao Y Shan Y Liu B Li Y Zhao L. MiR-106b and miR-93 regulate cell progression by suppression of PTEN via PI3K/Akt pathway in breast cancer. Cell Death Dis. (2017) 8:e2796. 10.1038/cddis.2017.119 28518139 PMC5520687

[44] Mehlich D Garbicz F Wlodarski PK. The emerging roles of the polycistronic miR-106b approximately 25 cluster in cancer—A comprehensive review. Biomed Pharmacother. (2018) 107:1183–95. 10.1016/j.biopha.2018.08.097 30257332

[45] Song HM Luo Y Li DF Wei CK Hua KY Song JL. MicroRNA-96 plays an oncogenic role by targeting FOXO1 and regulating AKT/FOXO1/Bim pathway in papillary thyroid carcinoma cells. Int J Clin Exp Pathol. (2015) 8:9889–900. 26617698 PMC4637783

[46] Hong Y Liang H Uzair Ur R Wang Y Zhang W Zhou Y. miR-96 promotes cell proliferation, migration and invasion by targeting PTPN9 in breast cancer. Sci Rep. (2016) 6:37421. 10.1038/srep37421 27857177 PMC5114647

[47] Song CL Zhang LJ Wang J Huang ZY Li X Wu MQ. High expression of microRNA-183/182/96 cluster as a prognostic biomarker for breast cancer. Sci Rep. (2016) 6. 10.1038/srep24502 27071841 PMC4829912

[48] Huang X Lv W Zhang JH Lu DL miR-96 functions as a tumor suppressor gene by targeting NUAK1 in pancreatic cancer. Int J Mol Med. (2014) 34:1599–605. 10.3892/ijmm.2014.1940 25242509

[49] Ress AL Stiegelbauer V Winter E Schwarzenbacher D Kiesslich T Lax S. MiR-96-5p influences cellular growth and is associated with poor survival in colorectal cancer patients. Mol Carcinogenesis. (2015) 54:1442–50. 10.1002/mc.22218 25256312

[50] Boren T Xiong Y Hakam A Wenham R Apte S Wei ZZ. MicroRNAs and their target messenger RNAs associated with endometrial carcinogenesis. Gynecol Oncol. (2008) 110:206–15. 10.1016/j.ygyno.2008.03.023 18499237

[51] Prado MM Frampton AE Giovannetti E Stebbing J Castellano L Krell J Investigating miRNA-mRNA regulatory networks using crosslinking immunoprecipitation methods for biomarker and target discovery in cancer. Expert Rev Mol Diagnostics. (2016) 16:1155–62. 10.1080/14737159.2016.1239532 27784183

[52] Luo D Wilson JM Harvel N Liu J Pei L Huang S. A systematic evaluation of miRNA:mRNA interactions involved in the migration and invasion of breast cancer cells. J Transl Med. (2013) 11:57. 10.1186/1479-5876-11-57 23497265 PMC3599769

[53] Zhou X Xu X Wang J Lin J Chen W. Identifying miRNA/mRNA negative regulation pairs in colorectal cancer. Sci Rep. (2015) 5:12995. 10.1038/srep12995 26269151 PMC4534763

[54] Andres-Leon E Cases I Alonso S Rojas AM. Novel miRNA-mRNA interactions conserved in essential cancer pathways. Sci Rep. (2017) 7:46101. 10.1038/srep46101 28387377 PMC5384238

[55] Santamaria PG Moreno-Bueno G Portillo F Cano A. EMT: present and future in clinical oncology. Mol Oncol. (2017) 11:718–38. 10.1002/1878-0261.12091 28590039 PMC5496494

[56] Baserga R. The contradictions of the insulin-like growth factor 1 receptor. Oncogene. (2000) 19:5574–81. 10.1038/sj.onc.1203854 11114737

[57] Tomasetti M Nocchi L Staffolani S Manzella N Amati M Goodwin J. MicroRNA-126 suppresses mesothelioma malignancy by targeting IRS1 and interfering with the mitochondrial function. Antioxid Redox Signal. (2014) 21:2109–25. 10.1089/ars.2013.5215 24444362 PMC4215384

[58] Ye JJ Cao J. MicroRNAs in colorectal cancer as markers and targets: recent advances. World J Gastroenterol. (2014) 20:4288–99. 10.3748/wjg.v20.i15.4288 24764666 PMC3989964

[59] Yu Y Li X Liu L Chai J Haijun Z Chu W. miR-628 promotes burn-induced skeletal muscle atrophy via targeting IRS1. Int J Biol Sci. (2016) 12:1213–24. 10.7150/ijbs.15496 27766036 PMC5069443

[60] Xu Y Luo S Liu Y Li J Lu Y Jia Z. Integrated gene network analysis and text mining revealing PIK3R1 regulated by miR-127 in human bladder cancer. Eur J Med Res. (2013) 18:29. 10.1186/2047-783X-18-29 24004856 PMC3766679

[61] Yan LX Liu YH Xiang JW Wu QN Xu LB Luo XL. PIK3R1 targeting by miR-21 suppresses tumor cell migration and invasion by reducing PI3K/AKT signaling and reversing EMT, and predicts clinical outcome of breast cancer. Int J Oncol. (2016) 48:471–84. 10.3892/ijo.2015.3287 26676464 PMC4725461

[62] Kim S Lee E Jung J Lee JW Kim HJ Kim J. microRNA-155 positively regulates glucose metabolism via PIK3R1-FOXO3a-cMYC axis in breast cancer. Oncogene. (2018) 37:2982–91. 10.1038/s41388-018-0124-4 29527004 PMC5978802

[63] Fruman DA Chiu H Hopkins BD Bagrodia S Cantley LC Abraham RT. The PI3K pathway in human disease. Cell. (2017) 170:605–35. 10.1016/j.cell.2017.07.029 28802037 PMC5726441

[64] Murphy-Ullrich JE Mosher DF. Localization of thrombospondin in clots formed *in situ*. Blood. (1985) 66:1098–104. 3902120

[65] Schultzcherry S Ribeiro S Gentry L Murphyullrich JE Thrombospondin binds and activates the small and large forms of latent transforming growth-factor-beta in a chemically-defined system. J Biol Chem. (1994) 269:26775–82. 7929413

[66] Bornstein P. Thrombospondins function as regulators of angiogenesis. J Cell Commun Signal. (2009) 3:189–200. 10.1007/s12079-009-0060-8 19798599 PMC2778581

[67] Dogar AM Semplicio G Guennewig B Hall J. Multiple microRNAs derived from chemically synthesized precursors regulate thrombospondin 1 expression. Nucleic Acid Ther. (2014) 24:149–59. 10.1089/nat.2013.0467 24444023 PMC3962651

[68] Williams CM Engler AJ Slone RD Galante LL Schwarzbauer JE. Fibronectin expression modulates mammary epithelial cell proliferation during acinar differentiation. Cancer Res. (2008) 68:3185–92. 10.1158/0008-5472.CAN-07-2673 18451144 PMC2748963

[69] Yen CY Huang CY Hou MF Yang YH Chang CH Huang HW. Evaluating the performance of fibronectin 1 (FN1), integrin alpha4beta1 (ITGA4), syndecan-2 (SDC2), and glycoprotein CD44 as the potential biomarkers of oral squamous cell carcinoma (OSCC). Biomarkers. (2013) 18:63–72. 10.3109/1354750x.2012.737025 23116545

[70] Gong J Wang ZX Liu ZY miRNA-1271 inhibits cell proliferation in neuroglioma by targeting fibronectin 1. Mol Med Rep. (2017) 16:143–50. 10.3892/mmr.2017.6610 28535003 PMC5482146

[71] Ding Y Pan YH Liu S Jiang F Jiao JB. Elevation of MiR-9-3p suppresses the epithelial-mesenchymal transition of nasopharyngeal carcinoma cells via down-regulating FN1, ITGB1 and ITGAV. Cancer Biol Ther. (2017) 18:414–24. 10.1080/15384047.2017.1323585 28613134 PMC5536934

[72] Gunadi B Budi NYP Kalim AS Santiko W Musthofa FD Iskandar K. Aberrant expressions of miRNA-206 target, FN1, in multifactorial Hirschsprung disease. Orphanet J Rare Dis. (2019) 14:5. 10.1186/s13023-018-0973-5 30616633 PMC6323865

