# Supplementary material for: MicroRNA and mRNA Interaction Network Regulates the Malignant Transformation of Human Bronchial Epithelial Cells Induced by Cigarette Smoke
Source: Front Oncol. 2019 Oct 9;9:1029. doi: 10.3389/fonc.2019.01029 (PMC6794608; doi:10.3389/fonc.2019.01029)
Supplement: Supplementary file 1 [file Table_1.DOCX]

**Supplementary Table S1 Differentially expressed miRNAs between S30 cells and normal BEAS-2B cells**

| miRNA | log2FoldChange | pvalue | Significance |
| --- | --- | --- | --- |
| hsa-miR-106b-5p | 1.902118973 | 1.27E-12 | UP |
| hsa-miR-589-5p | 1.312257952 | 1.59E-08 | UP |
| hsa-miR-96-5p | 1.51096735 | 3.66E-07 | UP |
| hsa-miR-361-3p | -1.170255267 | 2.76E-06 | DOWN |
| hsa-miR-181a-5p | -1.189453692 | 3.22E-06 | DOWN |
| hsa-miR-378a-3p | -1.01999677 | 3.88E-06 | DOWN |
| hsa-miR-29c-3p | -9.411700868 | 4.94E-06 | DOWN |
| hsa-miR-29c-5p | 8.979923922 | 1.68E-05 | UP |
| hsa-miR-149-5p | 1.624618275 | 2.47E-05 | UP |
| hsa-miR-190a-5p | -8.724605761 | 3.74E-05 | DOWN |
| hsa-miR-502-3p | -1.132689221 | 1.38E-04 | DOWN |
| hsa-miR-582-3p | -8.279740194 | 1.40E-04 | DOWN |
| hsa-miR-9-5p | -1.52962769 | 6.28E-04 | DOWN |
| hsa-miR-585-3p | 7.359253904 | 8.78E-04 | UP |
| hsa-miR-548j-5p | 6.95106939 | 1.90E-03 | UP |
| hsa-miR-381-3p | 6.885898027 | 2.06E-03 | UP |
| hsa-miR-3158-3p | 1.1238933 | 2.31E-03 | UP |
| hsa-miR-660-5p | -1.122578364 | 2.66E-03 | DOWN |
| hsa-miR-4684-3p | 1.482363237 | 3.56E-03 | UP |
| hsa-miR-4473 | 6.505509425 | 4.70E-03 | UP |
| hsa-miR-548az-5p | 6.348190842 | 4.96E-03 | UP |
| hsa-miR-4661-5p | 6.349919953 | 5.05E-03 | UP |
| hsa-miR-597-5p | 6.314271059 | 5.27E-03 | UP |
| hsa-miR-1287-5p | -1.95507456 | 5.74E-03 | DOWN |
| hsa-miR-641 | 6.137721959 | 6.77E-03 | UP |
| hsa-miR-93-5p | 1.239113798 | 7.36E-03 | UP |
| hsa-miR-3173-5p | 5.992933516 | 8.28E-03 | UP |
| hsa-miR-627-3p | 6.011979485 | 8.37E-03 | UP |
| hsa-miR-369-3p | 5.982714076 | 8.46E-03 | UP |
| hsa-miR-892c-3p | 5.736518231 | 1.18E-02 | UP |
| hsa-miR-4755-5p | 5.769406782 | 1.21E-02 | UP |
| hsa-miR-195-5p | -6.212061538 | 1.38E-02 | DOWN |
| hsa-miR-1-3p | -1.1781113 | 1.53E-02 | DOWN |
| hsa-miR-122-5p | 1.285383769 | 1.53E-02 | UP |
| hsa-miR-504-5p | -6.078287559 | 1.60E-02 | DOWN |
| hsa-miR-4504 | 5.511618295 | 1.63E-02 | UP |
| hsa-miR-548ay-3p | 5.37623956 | 1.98E-02 | UP |
| hsa-miR-548l | 5.210589562 | 2.25E-02 | UP |
| hsa-miR-2682-5p | 1.568665731 | 2.97E-02 | UP |
| hsa-miR-362-5p | -1.569477275 | 3.22E-02 | DOWN |
| hsa-miR-514a-3p | 2.15708795 | 3.40E-02 | UP |
| hsa-miR-3688-3p | 5.220236168 | 4.65E-02 | UP |

**Supplementary Table S2 The negatively correlated miRNA-mRNA pairs in S30 cells**

| miRNAs | DEGs | Gene Symbol |
| --- | --- | --- |
| UP | DOWN | FAM129A, TGM2, EPAS1, L1CAM, STC1, TNS1, IRS1, NT5E, SQSTM1, PREX1, COL13A1, ABCA1, TRIB3, PAPPA, PPARA, TXNIP, SESN2, NAV2, CHST10, CCDC71L, CORO2B, SH3PXD2B, PAG1, PKD1, POU2F2, FOXN3, TTYH3, KLF9, HDAC9, TMCC3, RAB27B, F2R, LAMA3, NANOS1, CDK18, MAGI1, ADAM22, SLC6A9, PSD3, RAVER2, TMTC1, PIK3R1, CAMK2N1, SOGA1, S1PR1, ZNF697, ST3GAL1, CDYL2, OSBPL5, TMEM25, PDE1C, MAP3K1, CHST1, SLC25A27, SPESP1, PPP1R3B, BNC2, ITPR1, CXCL6, SPRY4, SLC2A3, SCAMP5, NPAS2, OTUD1, ANKRD29, GPR135, CREBRF, L3MBTL3, TIMP4, SMAD7 |
| DOWN | UP | ALDH1A3, MFAP5, AK4, SVEP1, CLU, CSF1, SH2D4A, FN1, SGK1, TUFT1, ELL2, THBS1, UBL3, KCNMA1, DNAJB4, TXNDC17, TUBA4A, CCL2, ARPC3, TSPAN9, OSTF1, AP1S3, NETO1, C18ORF21, C4ORF26 |
